# Supplementary material for: First observations of ovary regeneration in an amphipod, Ampelisca eschrichtii Krøyer, 1842
Source: PeerJ. 2022 Mar 10;10:e12950. doi: 10.7717/peerj.12950 (PMC8918206; doi:10.7717/peerj.12950)
Supplement: Supplemental Information 1 — (M_ibm) – ecs on the intestinal basal membrane of immature 13.5 mm male. (M_test) – mcs in the germinal zone of immature 13.5 mm male testes. (Fnorm_ibm) – ecs on the intestinal basal membrane in the anterior part of the body in a female with normal ovaries. (Fatr_imb) – ecs on the intestinal basal membrane in the anterior part of the body in a female with ovarian atrophy. (Freg_nest) – ecs in the “nests” on the intestinal basal membrane of a female with regenerating ovaries. (Freg_migr) – putatively migrating ecs outside the intestinal basal membrane in the female with regenerating ovaries. (Freg_gz) – mcs in the germinal zone of a female with regenerating ovaries. (Freg_ibm) – ecs on the intestinal basal membrane opposite the germinal zone in a female with regenerating ovaries. [file peerj-10-12950-s001.docx]

| **Cell Source** | **M_imb** | **M_test** | **Fnorm_ibm** | **Fatr_ibm** | **Freq_nest** | **Freq_migr** | **Freq_gz** | **Freq_ibm** |
| --- | --- | --- | --- | --- | --- | --- | --- | --- |
| **Ave (µm^3^)** | 263.1 | 362.6 | 469.9 | 527.9 | 1399.7 | 2381.3 | 2510.8 | 3597.1 |
|  |  |  |  |  |  |  |  |  |
|  |  |  |  |  |  |  |  |  |
|  |  |  |  |  |  |  |  |  |
|  |  |  |  |  |  |  |  |  |
| **STD** | 85.9 | 100.8 | 161.5 | 209.7 | 650.4 | 1058.9 | 917.0 | 2041.4 |
| **N** | 10 | 10 | 20 | 20 | 16 | 10 | 8 | 17 |
| **SEM** | 27.2 | 31.9 | 36.1 | 46.9 | 162.6 | 334.8 | 324.2 | 495.1 |
