# Supplementary figures and images for: First observations of ovary regeneration in an amphipod, Ampelisca eschrichtii Krøyer, 1842
Source: PeerJ. 2022 Mar 10;10:e12950. doi: 10.7717/peerj.12950 (PMC8918206; doi:10.7717/peerj.12950)

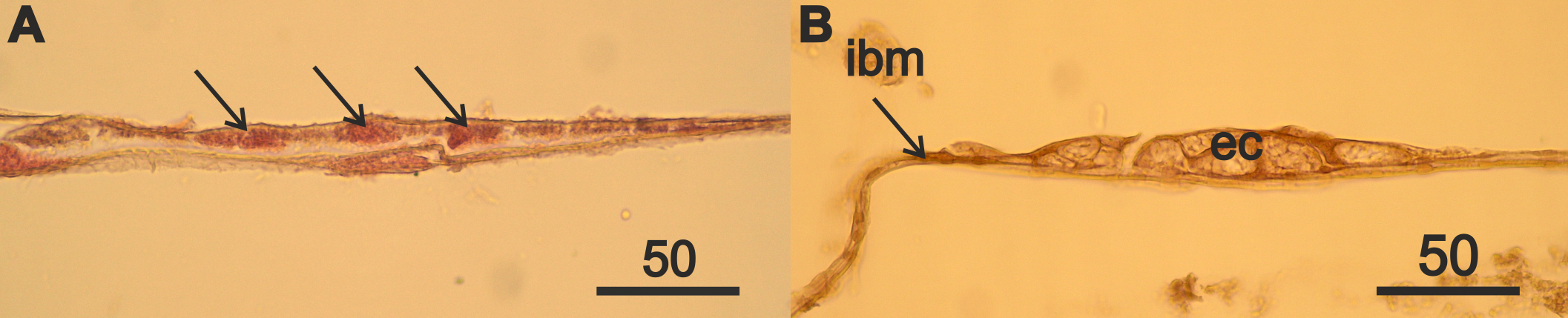

Supplement: Supplemental Information 2 — (A) the cells of the old mesoderm (arrows) in atrophied ovaries. (B) nest of eosinophilic cells (ec) on the intestinal basal membrane (ibm) opposite the atrophied anterior part of ovaries. Scales are in µm. [file peerj-10-12950-s002.png]

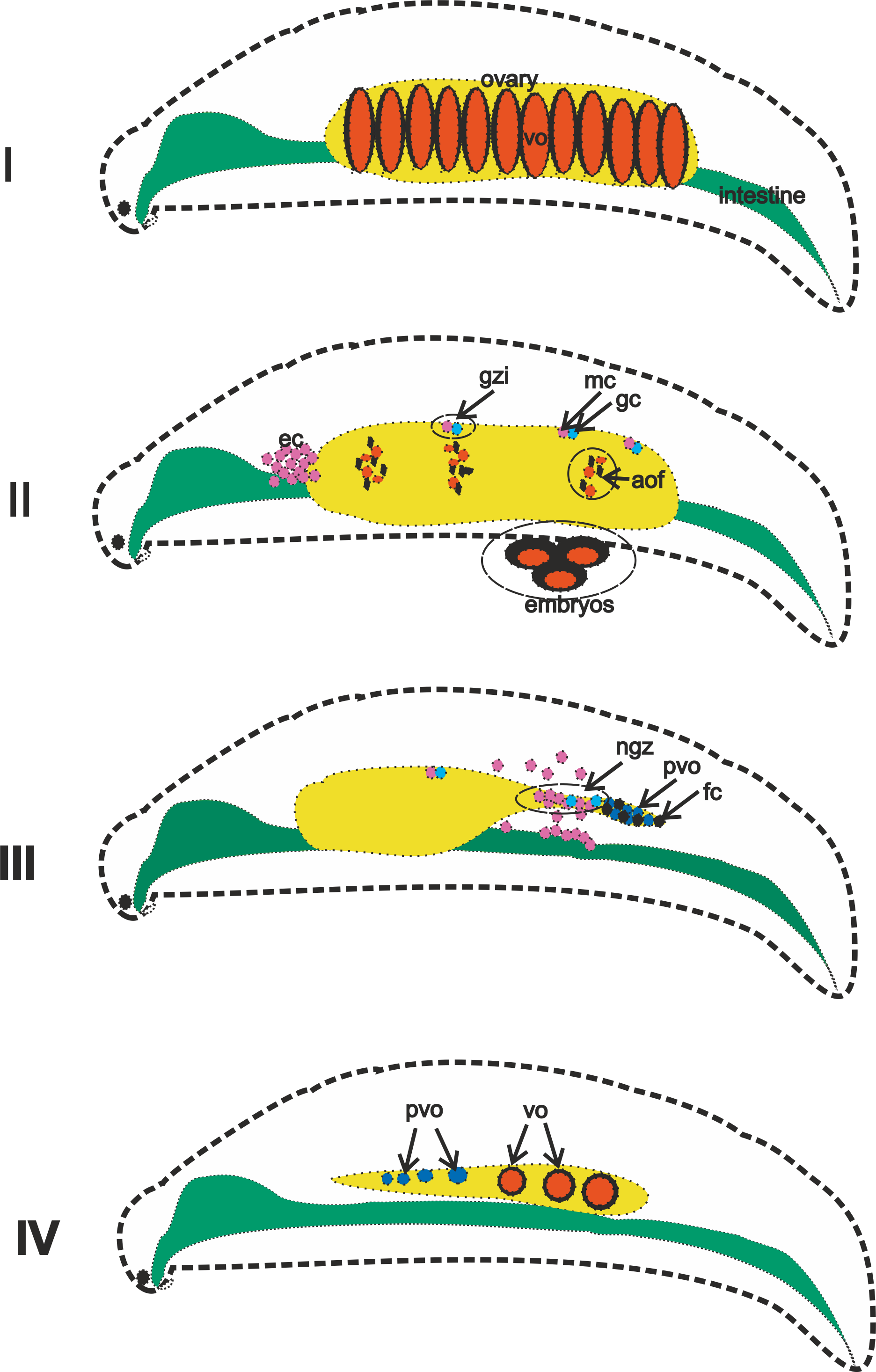

Supplement: Supplemental Information 3 — (I) Ovaries bearing first generation vitelogenic oocytes (vo). (II) vo of the first generation enter the marsupium where they are fertilized to become embryos, vo of the second generation are resorbed (= atresia of the ovarian follicles, aof), but the islets of the germinal zone (gzi) containing germline (gc) and mesodermal cells (mc) remain, while eosinophilic cells (ec) appear in masses in the body anterior to the ovaries. (III) Eosinophilic cells that migrate from the intestinal wall and the remaining germ cells forming a new germinal zone (ngz) that produces previtellogenic oocytes (pvo) and follicular epithelial cells (fc). (IV) Sequential transformation from previtellogenic oocytes to vitellogenic oocytes as ovarian regeneration progresses from posterior to anterior. [file peerj-10-12950-s003.png]
